# Fungus-mediated synthesis of Se-BiO-CuO multimetallic nanoparticles as a potential alternative antimicrobial against ESBL-producing *Escherichia coli* of veterinary origin

**DOI:** 10.3389/fcimb.2024.1301351

**Published:** 2024-03-22

**Authors:** Rida Rasheed, Bushra Uzair, Abida Raza, Reem Binsuwaidan, Nawaf Alshammari

**Affiliations:** ^1^ Department of Biological Sciences, International Islamic University, Islamabad, Pakistan; ^2^ National Center of Industrial Biotechnology, Pir Mehr Ali Shah Arid Agriculture University, Rawalpindi, Pakistan; ^3^ Department of Pharmaceutical Sciences, College of Pharmacy, Princess Nourah bint Abdulrahman University, Riyadh, Saudi Arabia; ^4^ Department of Biology, College of Science, University of Hail, Hail, Saudi Arabia

**Keywords:** *Talaromyces haitouensis*, Se-BiO-CuO-MMNPs, the antibacterial potential of MMNPs, swimming motility assay, cytoplasmic efflux analysis, biocompatibility study

## Abstract

Bacterial infections emerge as a significant contributor to mortality and morbidity worldwide. Emerging extended-spectrum β-lactamase (ESBL) *Escherichia coli* strains provide a greater risk of bacteremia and mortality, are increasingly resistant to antibiotics, and are a major producer of ESBLs. *E. coli* bacteremia-linked mastitis is one of the most common bacterial diseases in animals, which can affect the quality of the milk and damage organ functions. There is an elevated menace of treatment failure and recurrence of *E. coli* bacteremia necessitating the adoption of rigorous alternative treatment approaches. In this study, Se-Boil-CuO multimetallic nanoparticles (MMNPs) were synthesized as an alternate treatment from *Talaromyces haitouensis* extract, and their efficiency in treating ESBL *E. coli* was confirmed using standard antimicrobial assays. Scanning electron microscopy, UV–visible spectroscopy, and dynamic light scattering were used to validate and characterize the mycosynthesized Se-BiO-CuO MMNPs. UV–visible spectra of Se-BiO-CuO MMNPs showed absorption peak bands at 570, 376, and 290 nm, respectively. The average diameters of the amorphous-shaped Se-BiO-CuO MMNPs synthesized by *T. haitouensis* extract were approximately 66–80 nm, respectively. Se-BiO-CuO MMNPs (100 μg/mL) showed a maximal inhibition zone of 18.33 ± 0.57 mm against *E. coli*. Se-BiO-CuO MMNPs also exhibited a deleterious impact on *E. coli* killing kinetics, biofilm formation, swimming motility, efflux of cellular components, and membrane integrity. The hemolysis assay also confirms the biocompatibility of Se-BiO-CuO MMNPs at the minimum inhibitory concentration (MIC) range. Our findings suggest that Se-BiO-CuO MMNPs may serve as a potential substitute for ESBL *E. coli* bacteremia.

## Introduction

The worldwide increase in bacterial diseases poses a significant risk to both human and animal health (Abdelwahab et al., 2022). The infectious diseases associated with Gram-negative pathogens are a major concern and the failure of antibiotics to effectively eliminate pathogens represents one of the most critical challenges (Rajivgandhi et al., 2022). *Escherichia coli* infection is the most common cause of mastitis in the livestock sector around the globe. Mastitis is the most frequent disease in dairy cattle and is known to have an adverse effect on udder health and animal welfare. It also results in considerable financial losses for dairy growers (Cobirka et al., 2020). When bacteria from infected body organs or milk enter the bloodstream, a disease known as bacteremia damages the function of the body organs and may even lead to fatalities (Abegewi et al., 2022). *E. coli* (*Enterobacteriaceae* family) is the dominant and leading cause of bacteremia followed by *Streptococcus* spp. and *Klebsiella* spp (El-Mohandes et al., 2022; Johnson et al., 2015). Bacteremia has a devastating effect on the economy, patient morbidity, and mortality (Thaden et al., 2017). The emergence of antimicrobial-resistant pathogens particularly the extended-spectrum β-lactamase (ESBL)-producing *E. coli* has had a substantial impact on the treatment of infections (Mamani Huarani et al., 2019). Inhibition of drug uptake, modifying the drug target, and protein synthesis are some of the mechanisms through which bacterial cells reduce the effectiveness of antibiotics and develop resistance (Chatterjee et al., 2023). Bacterial communication by both transcription and quorum sensing is also the leading cause of multidrug resistance. The β-lactamase enzyme hydrolyzes the β-lactam drugs, which inhibit the drug efficacy and intercept cell lysis within the bacterial cell. ESBL-positive *E. coli* is also included in the list of pathogens that require alternative treatment options (Rajivgandhi et al., 2019d). β-lactam antibiotics have been regarded as the preferred treatment for ESBL infections. The efficacy of antibiotics within the *Enterobacteriaceae* family is in jeopardy due to the resistance that poses a significant challenge to the effectiveness of these antibiotics (Kettani Halabi et al., 2021). Currently, there are no vaccines available against *E. coli* bacteremia (Huttner et al., 2017). There is an increased risk of treatment failure and recurrence (Friedman et al., 2016) due to more than 55% of mastitis-causing ESBL *E. coli* bacterial resistance to antimicrobials (Krebs et al., 2023a).

Presently, green nanotechnology has gained a potential interest in scientific communities (Mohammadinejad et al., 2019). Various emerging nanomaterials with exceptional and distinctive qualities are undoubtedly a groundbreaking technique to address issues with multidrug resistance, efficient drug delivery, and advanced medical treatments, but this field still needs substantial research. Metallic nanoparticles contain unique and remarkable physicochemical and biological properties (Nasrollahzadeh et al., 2020). The most intriguing solutions against multidrug resistance are produced by combining these nanomaterials. In such amalgamations [multimetallic nanoparticles (MMNP)], they typically demonstrate stronger characteristics that differ from those of their components (Dobrucka, 2020). MMNPs have drawn significant interest from researchers because of their adaptable surface modifications and biocompatibility like amplified catalytic activity, robust antibacterial effects, improved drug encapsulation efficiency, numerous morphologies, extremely sensitive detection, and good stability (Basavegowda and Baek, 2021; Liu et al., 2012).

The synergistic effect caused by the two or three metals present in the bimetallic and trimetallic NPs is the cause of these promising features. Additionally, owing to the presence of multiple metals, multi-metal oxide nanoparticles can have a variety of morphologies and structures (Zaleska-Medynska et al., 2016). These MMNPs demonstrate potential antibacterial characteristics with quick and accurate bacterial identification. Pathogens can seldom build resistance against metal and MMO-NPs in particular because they damage bacterial membranes and produce reactive oxygen species (ROS) that act against bacterial cells (Lemire et al., 2013). Nonetheless, extensive work done on only monometallic, bimetallic, and a very small number of trimetallic NPs has been reported to date for their antibacterial activities (Basavegowda and Baek, 2021; Okkeh et al., 2021). Literature reported various methods of synthesizing nanoparticles including chemical, green synthesis, hydrothermal, mechanical alloying, co-precipitation, micro-emulsion, electrochemical, sol-gel, lithography, laser ablation, microwave (MW) irradiation, and gas-phase condensation (Sharma et al., 2017).

Biogenic synthesis of nanoparticles attracted scientist’s interest currently because of its secure, affordable, and simple scale-up approach (Saad et al., 2018). However, fungi produce metal nanoparticles more advantageously because of various unique properties like numerous reducing agents (enzymes and proteins), simple, biomass processing, easy scaling up, and fast growth (Fouda et al., 2020; Rai et al., 2021). Additionally, the mycosynthesized nanoparticles green approach is chosen over other biological methods from an economic perspective (Soliman et al., 2021). Numerous metal oxide nanoparticles have so far been stated using fungal cell extract including CeO, CuO, and ZnO (Mohamed et al., 2019; Gopinath et al., 2015). Because of their non-toxicity and biocompatibility, such nanoparticles are typically acknowledged as benign materials by the US Food and Drug Administration (Zhong et al., 2018; Mohamed et al., 2021). Moreover, the literature study highlighted a few examples of mycosynthesized MMNPs (Dobrucka, 2020).

In this research study, we report the extracellular synthesis of Se-BiO-CuO MMNPs by the fungus *Talaromyces haitouensis* for the first time. To the best of our knowledge, no prior study of the synthesized Se-BiO-CuO MMNPs has been reported. These nanoparticles were characterized by SEM, XRD, and ultraviolet–visible spectroscopy. The *in vitro* biological and killing kinetic activities, biofilm analysis, cytoplasmic efflux, and swimming motility of Se-BiO-CuO MMNPs were also examined.

## Experimental design

### Materials and methods

The following materials were used in the study: chitosan, copper sulfate pentahydrate, bismuth nitrate, sodium selenite, glacial acetic acid, Mueller–Hinton broth (MHB), Mueller–Hinton agar (MHA), Sabouraud dextrose agar (SDA), Sabouraud dextrose broth (SDB), dimethyl sulfoxide (DMSO), and Triton X-100 (Sigma-Aldrich). During all the research, double-distilled water (DDW) was used. The International Islamic University and Nanomedicine Research Laboratory NILORE Islamabad provided plastic, glassware, and other chemicals.

### Isolation of bacterial strain

The jugular vein was examined by the veterinarian to collect blood samples. Thus, 30% ethanol was used to disinfect the skin of the cow suffering from mastitis. Blood samples were taken with the help of a syringe (20 mL) (Krebs et al., 2023b). Subsequently, for the isolation of ESBL, *E. coli* sample was promptly placed into a flask containing brain heart infusion (BHI) broth, cultured on selected media, and incubated for 24 h at 37°C. To isolate the pure culture, the plates were sub-cultured on MacConkey agar for the isolation of *E. coli* (Shukla et al., 2015).

### Screening of ESBL

The screening of ESBL *E. coli* was confirmed by the method already reported in literature (Rajivgandhi et al., 2019b). In an antibiotic susceptibility test, isolates resistant to ceftazidime, cefotaxime, and ceftriaxone were identified as potential ESBL producers (CLSI guidelines). The augmentin disc was placed in the center and the aztrenam disc, together with three third-generation cephalosporin discs (ceftriaxone, cefotaxime, and ceftazidime), were positioned at a 20-mm spacing and placed in the incubator by the double-disc synergistic test. The zone of inhibition was observed, and the maximum zone of inhibition and the synergy between amoxicillin/clavulanic acid and cefotaxime or ceftazidime were an indicator of ESBL positivity. Rajivgandhi et al. (2018) reported that majority of Gram-negative bacteria strains notably *E. coli* and *K. pneumoniae*, isolated from wounds, pus, and blood, produced increased amounts of ESBLs. These bacteria were highly resistant to available antibiotics. Furthermore, within the *Enterobacteriaceae* family, the majority of the isolates were categorized as MDR with a much higher rate of ESBL synthesis.

### Isolation and identification of fungal strain

#### Morphological and molecular identification

*T. haitouensis*, employed in this research work for the synthesis of MMNPs, was isolated from rhizospheric soil. It was cultured on SDA medium at 28°C and subsequently stored at 4°C. The fungus was characterized through an assessment of its colony morphology and micro-morphological traits, Additionally, it was identified using a molecular approach involving the analysis of the Internal Transcribed Spacer (ITS) gene sequence. The micro-morphological characteristics of the strain were examined after staining with lacto phenol cotton blue and subsequent observation under a light microscope (Komal et al., 2020). The genomic DNA was isolated using the cetyltrimethylammonium bromide (CTAB) technique. The fungal strain was incubated in 50 mL of Sabouraud broth for 3 days at 28°C, and the biomass was then collected. The fungus genomic DNA was isolated using a TaKaRa DNA extraction kit. The ITS region of the fungus was amplified by PCR using the ITS forward primer (5′TCCGTAGGTGAACCTGCGG3′) and reverse primer (5′TCCTCCGCTTATTGATATGC3′). BLAST software was performed to conduct homology research on the NCBI website. The aqueous extract of *T. haitouensis* was stored at 4°C for the MMNP synthesis (Singh et al., 2020).

#### Preparation of *Talaromyces haitouensis* extract

*T. haitouensis* was freshly inoculated in a potato dextrose broth in a flask. The flask was incubated on an orbital shaker at 200 rpm (5 days, 28 ± 2°C). The fungal biomass was harvested through Whatman No. 1 filter paper, and later thoroughly washed with deionized water to remove the media components from the biomass. Typically, 15 g of fresh and clean biomass was taken into an Erlenmeyer flask containing 100 mL of deionized water and the flask was incubated at 30°C for 3 days and agitated at 200 rpm. The cell-free filtrate was obtained using Whatman No. 1 filter paper at the end and stored for further applications (Rani et al., 2017).

#### GC-MS analysis of fungal extract

GC-MS analysis of *T. haitouensis* extract was performed using a GC-MS analyzer (BRUKER SCION 436-GC SQ, USA). The sample was examined by capillary column (30 m length, 0.25 mm column inside diameter with 0.25 µm film coating) with He gas as the carrier with a flow rate of 1 mL/min. Fungal extract in a volume of 1 L was injected into a column with an inlet temperature of 280°C. The MS of the compounds found in the fungal extract was obtained by electron ionization at 70 eV. The extracted spectrum results were compared to the National Institute of Science and Technology (NIST) library’s database. The component names, molecular weights, and formulas were determined (Sharma et al., 2022).

#### Synthesis and purification of Se-BiO-CuO multimetallic nanoparticles

Typically, 50 mL of fungal cell-free filtrate was added to 1 mM solution of the CuSO_4_·5H_2_O, Bi(NO_3_)_3_·5H_2_O, and Na_2_SeO_3_(H_2_O)_5_ in 250-mL Erlenmeyer flasks. The reaction mixture’s temperature was set at 45°C, and it was aggressively stirred in the dark for 48 h. Any change in the solution color was used to monitor and confirm the formation of the Se-BiO-CuO MMNPs. The synthesized MMNPs were separated by centrifugation (15,000 rpm for 20 min) and rinsed with distilled water twice and then with 70% ethanol. The MMNPs were then allowed to dry at room temperature for 24 h before being lyophilized (FreeZone 6, Labconco, Kansas City, MO, USA). Then, nanoparticles were kept at ambient temperature and in darkness in amber-colored polypropylene tubes (Vaseghi et al., 2018).

#### Physicochemical characterization of multimetallic nanoparticles

A UV–vis absorption spectrophotometer (U-2900 UV-VIS Spectrophotometer – HITACHI High-Tech Science, Tokyo, Japan; λ = 200–1,100 nm) was used to monitor the synthesis of Se/Bi/Cu MMNPs in the 200- to 800-nm wavelength range. As a blank, the deionized water was used (Chowdhury et al., 2022).

Dynamic light scattering (DLS; Microtrac Nanotrac Wave II, PA, USA) analysis was done with a Zetasizer Nano ZS (Malvern Instruments) according to the already reported method (Chattopadhyay et al., 2012). A suspension (100 μg/mL) of the synthesized MMNPs was diluted in Milli-Q water, sonicated for 20 min at ambient temperature, and their size distribution was measured by dynamic fluctuations of light scattering intensity. All measurements were carried out in triplicate to obtain the average size of the MMNPs. Fourier transform infrared spectroscopy (FT-IR) was used to examine the functional characteristics of the green-synthesized Se-BiO-CuO MMNPs (Model JASCO-460 spectrometer) by the KBr compression method. The x-ray diffraction pattern was recorded by an x-ray diffractometer in the 2θ range from 20° to 80°. The average particle size of Se-BiO-CuO MMNPs was determined using the Debye–Scherrer formula (Chen et al., 2019). Field emission gun scanning electron microscopy (FEGSEM) was used to analyze the surface morphology of nanoparticles. In a nutshell, Se-BiO-CuO MMNPs (1 mg/mL) were dissolved in deionized water to obtain a uniform suspension achieved by sonication, and topographical images were taken (Das et al., 2017). The elemental makeup of a sample is ascertained using the FEGSEM-EDX (energy dispersive x-ray) analyzer. It was employed to identify the other elemental compositions of the particles as well as to validate the existence of bismuth, selenium, and copper in the particles. Mapping of the sample was also accomplished using pseudo-colors to represent the two-dimensional spatial energy distribution emissions of the elemental components (Chen et al., 2019).

#### Antibacterial potential of multimetallic nanoparticles

According to recommendations from the CLSI (Clinical and Laboratory Standards Institute guidelines), antimicrobial activities of Se-BiO-CuO MMNPs were evaluated. The agar well diffusion method was used to assess the antibacterial activity of Se-BiO-CuO MMNPs. The overnight bacterial suspension (1.5×10^8^ CFU/mL) was then disseminated uniformly on MHA plates using a sterile cotton ball and Se-BiO-CuO MMNP dipped discs were placed on media in Petri plates. After 24 h of incubation at 37°C, the zone of inhibition was gauged in mm (Huq and Akter, 2021).

The broth microdilution method was used to evaluate the minimum inhibitory concentration (MIC) of Se-BiO-CuO MMNPs against multidrug-resistant *E. coli* strain. A bacterial solution (100 μL) was disseminated in the 96-well plate that contained different concentrations of Se-BiO-CuO MMNPs. In each well, a total volume of 200 μL was adjusted and incubated for 24 h at ambient temperature (Gopinath et al., 2017). MIC was determined by measuring the absorbance at 600 nm using a microplate reader (Model FL ×800; Biotek, VT, USA). The inhibition growth percentage was demonstrated according to the reported literature (Singh et al., 2020).

### The action of Se-BiO-CuO MMNPs on the bacterial cell structure by SEM

Scanning electron microscopy (SEM) analysis revealed the deformations caused by the Se-BiO-CuO MMNPs against *E. coli*. A final bacterial concentration of 10^6^ CFU/mL was attained by diluting the overnight suspension. The bacterial solution treated with the MIC of MMNPs was agitated at 200 rpm for 24 h at 37°C while a solution without nanoparticles was used as control. The cultures were centrifuged after incubation and the supernatants were discarded. The bacterial pellet was treated with 50 μL of glutaraldehyde (2.5%) for 15 min and then washed with 30% ethanol. The samples were dried and then gold-coated for structural analysis under a scanning electron microscope (SEM, Hitachi S-3000N) (Al-Otibi et al., 2023).

### Killing kinetic study

The previously reported method was used to study the killing kinetic test of ESBL *E. coli* (1.5×10^8^ CFU/mL) against Se-BiO-CuO MMNPs. The absorbance of bacterial growth treated with MIC value at different time intervals was measured while positive control was without any Se-BiO-CuO MMNPs using a UV–vis spectrophotometer (U-2900 UV-VIS Spectrophotometer – HITACHI High-Tech Science, Tokyo, Japan; λ = 200–1,100 nm). Sequential OD measurements were used to graph the *E. coli* growth curves in GraphPad Prism (Das et al., 2017).

### Swimming motility assay

The swimming motility test of the *E. coli* strain was evaluated on semi-solid MHA. Se-BiO-CuO MMNPs at 100, 150, 300, 600, and 1,000 μg/mL concentrations were added to the soft agar medium. Then, 20 μL of *E. coli* suspension in the middle with or without MMNPs containing plates was dropped and incubated for 48 h at 37°C. To evaluate the swimming ability of the *E. coli* strain as a result of exposure to Se-BiO-CuO MMNPs, the migration diameter was measured. The experiment was performed in triplicate (Saeki et al., 2021).

### Cytoplasmic efflux analysis

The optical density of the solution at 260 nm demonstrated that, upon disruption to the cell membrane, chemicals (DNA and RNA) were liberated out of the cell. The 1,000 μL overnight bacterial inoculum with Se-BiO-CuO MMNPs (50, 100, 150, and 200 μg/mL) was cultivated at 37°C for 10 h. The bacteria and Se-BiO-CuO MMNPs were subsequently removed from the samples by centrifuging them at 6,000*g* for 10 min. Using a spectrophotometer (U-2900 UV-VIS Spectrophotometer – HITACHI High-Tech Science, Tokyo, Japan), the solution’s OD value was recorded at 260 nm ultraviolet light (Cai et al., 2018).

### Biofilm inhibition assay

The anti-biofilm activity of the Se-BiO-CuO MMNPs was evaluated using a 96-well microtiter plate according to the protocol already reported (Shehabeldine et al., 2023). To determine the outcomes of the biofilm studies, crystal violet staining was used on a static *E. coli* biofilm model. Mycosynthesized Se-BiO-CuO MMNPs in different concentrations were diluted into 96-well plates, supplemented with bacterial suspension (100 μL), and incubated at 37°C for 48 h. The wells were then washed twice with sterile distilled water followed by discarding supernatant before being stained for 20 min with 0.5% crystal violet. The samples were then again washed with distilled water before ethanol (95%) was added to each well. Absorbance was measured at 595 nm. The percentage of biofilm inhibition was calculated using the equation below (Fathil and Katas, 2023).


$$\text{Biofilm inhibition \%}=\frac{\text{OD}\left(\text{control}\right)-\text{OD}\left(\text{treated}\right)}{\text{OD}\left(\text{control}\right)}\times 100$$

To minimize the error rate, every trial was performed in triplicate and the photographs were assessed using a cell imager (Evos^®^R FL Cell Imaging System; Thermo Fisher Scientific, MA, USA).

### Biocompatibility analysis of Se-BiO-CuO MMNPs

The effect of Se-BiO-CuO MMNPs on red blood cells (RBCs) was assessed according to the protocol already reported (Nasar et al., 2022). From plasma, fresh RBCs were extracted by centrifugation for 10 min at 1,500 rpm. The erythrocytes were then washed and resuspended in phosphate-buffered saline at a cell concentration of 2% (v/v). In erythrocyte suspension, different concentrations of nanoparticles (50, 100, 150, 200, and 300 μg/mL) were combined. The negative control was PBS (pH 7.4), whereas the positive control was Triton X-100. The mixtures were centrifuged at 1,500 rpm (213×*g*) after 3 h at 37°C for 10 min to separate the RBCs. The supernatant has been retrieved and examined at 576 nm for dissolved hemoglobin.

### Statistical analysis

GraphPad Prism version 8.4.2 was used to analyze the data. The MIC values obtained for the Se-BiO-CuO MMNPs were statistically analyzed using the *t*-test and a 95% confidence interval. Statistical significance was defined as a *p*-value< 0.05.

## Results and discussion

### Isolation and confirmation of ESBL *E coli* strain

**Scheme 1:**
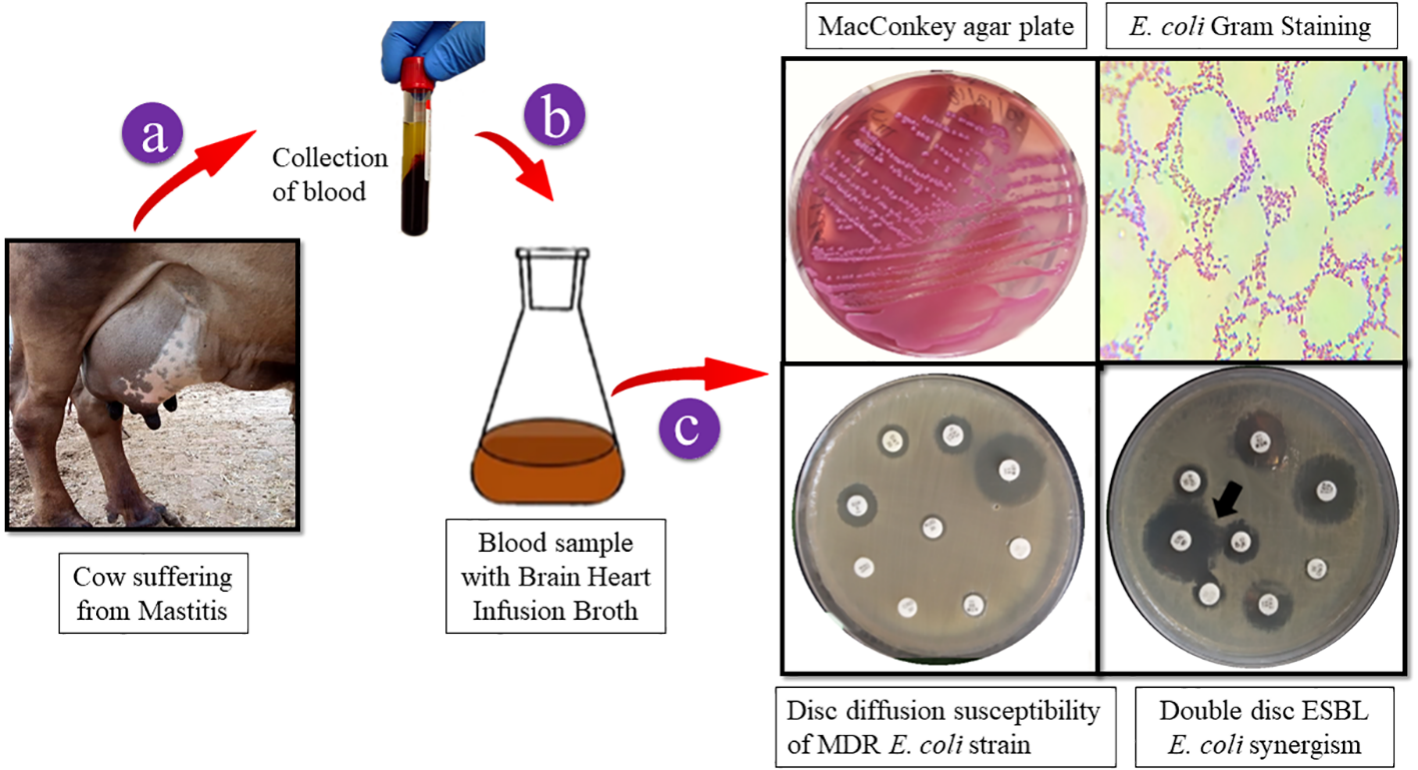
A graphic depicting the collection of cow blood samples. *E. coli* strain isolation on MacConkey agar. Gram-stained ESBL *E. coli* colonies were examined under a light microscope to reveal Gram-negative rods that ranged in color from red to pink. *E. coli* multidrug resistance was confirmed by disc diffusion and ESBL generation using the double-disc synergy method.

Samples in BHI broth were incubated and cultured on selective MacConkey agar and incubated for 24 h at 37°C. Following the culture, isolation, and examination of colony morphology, Gram staining was performed to identify the ESBL *E. coli*. On a MacConkey agar plate, the growth exhibited mucoid colonies with a faint rose-pink color (3–4 mm diameter). The double-disc synergy method was used to confirm the presence of ESBLs in *E. coli* (CLSI guidelines). Synergism was evident in ESBL-positive *E. coli* as shown in **Scheme 1**.

### Isolation and identification of fungi

The fungus *T. haitouensis* (GenBank accession no OP522007)

**Figure 1:**
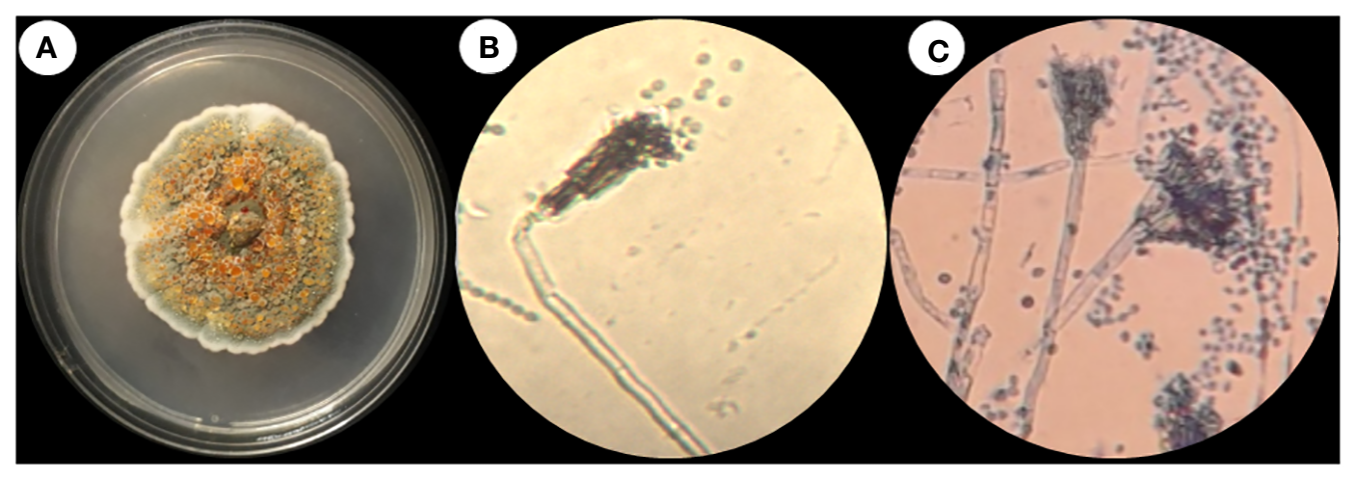
**(A)** *Talaromyces haitouensis* macroscopic colony morphology. **(B)** Microscopic morphology without staining **(C)** Microscopic morphology stained with lacto phenol cotton blue.

**Figure 2:**
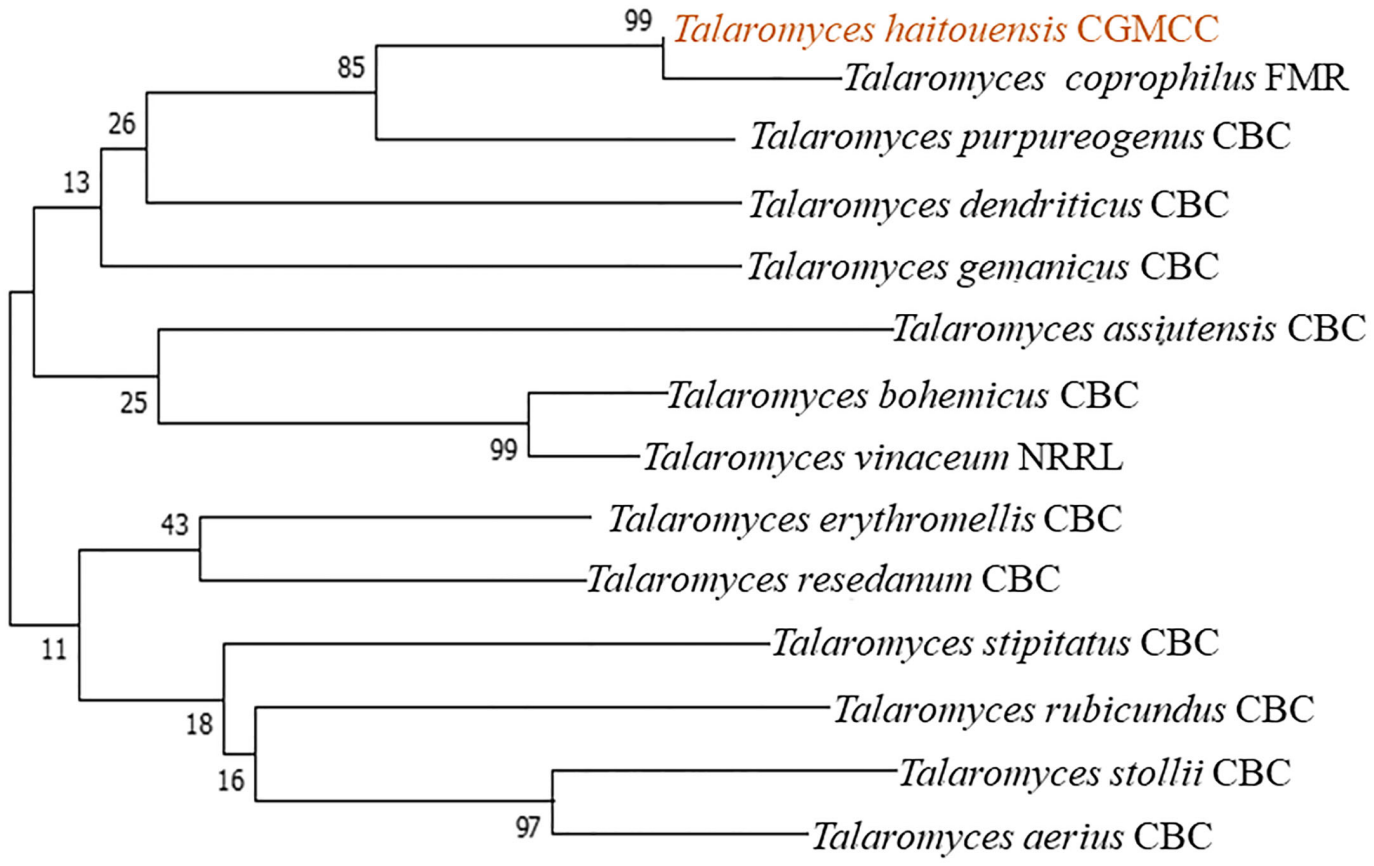
Taxonomic evolutionary relationships. The Neighbor-Joining approach was used to deduce the ancestral lineage. To calculate the evolutionary distances, the Maximum Composite Likelihood approach was used and the entire evolutionary analysis was carried out using MEGA7.

**Figure 3:**
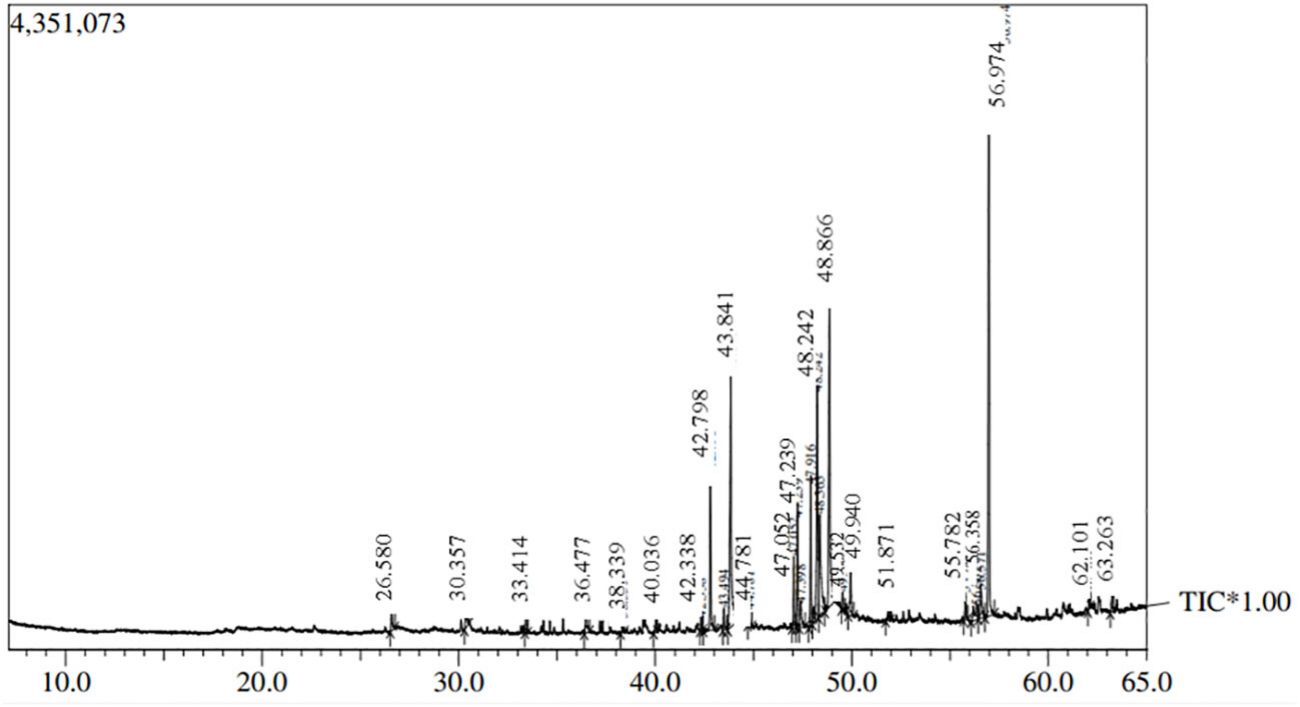
GC-MS chromatogram of *Talaromyces haitouensis* fungal extract.

Fungi were isolated from the rhizospheric soil of the National Agriculture Research Center Islamabad. A fungal isolate was selected for molecular identification using ITS sequencing following preliminary morphological identification as shown in **Figure 1**. The studied fungus demonstrated similarities to the genus *Talaromyces. Talaromyces* species are capable of producing a variety of antibacterial, antifungal anticancer secondary metabolites (Sun et al., 2020). To demonstrate the evolutionary link between the fungal strain used to synthesize MMNPs and closely related strains received from Gene Bank National Center for Biotechnology Information (NCBI), a phylogenetic tree was constructed as represented in **Figure 2**. The cell-free culture filtrate was stored and used for further studies. GC-MS analysis of *T. haitouensis* extract revealed the presence of 25 compounds when compared with the NIST Database. The GC-MS chromatogram is displayed in **Figure 3**. Major identified compounds were Trifluoroacetate (62.1), Ergostatriene (63.27), Palmitic acid (56.57), Phenanthrenecarboxylic acid (49.9), Phenol (30.4), and Oleic acid (47.24), among others.

### Biosynthesis and nanoparticle characterization

**Scheme 2:**
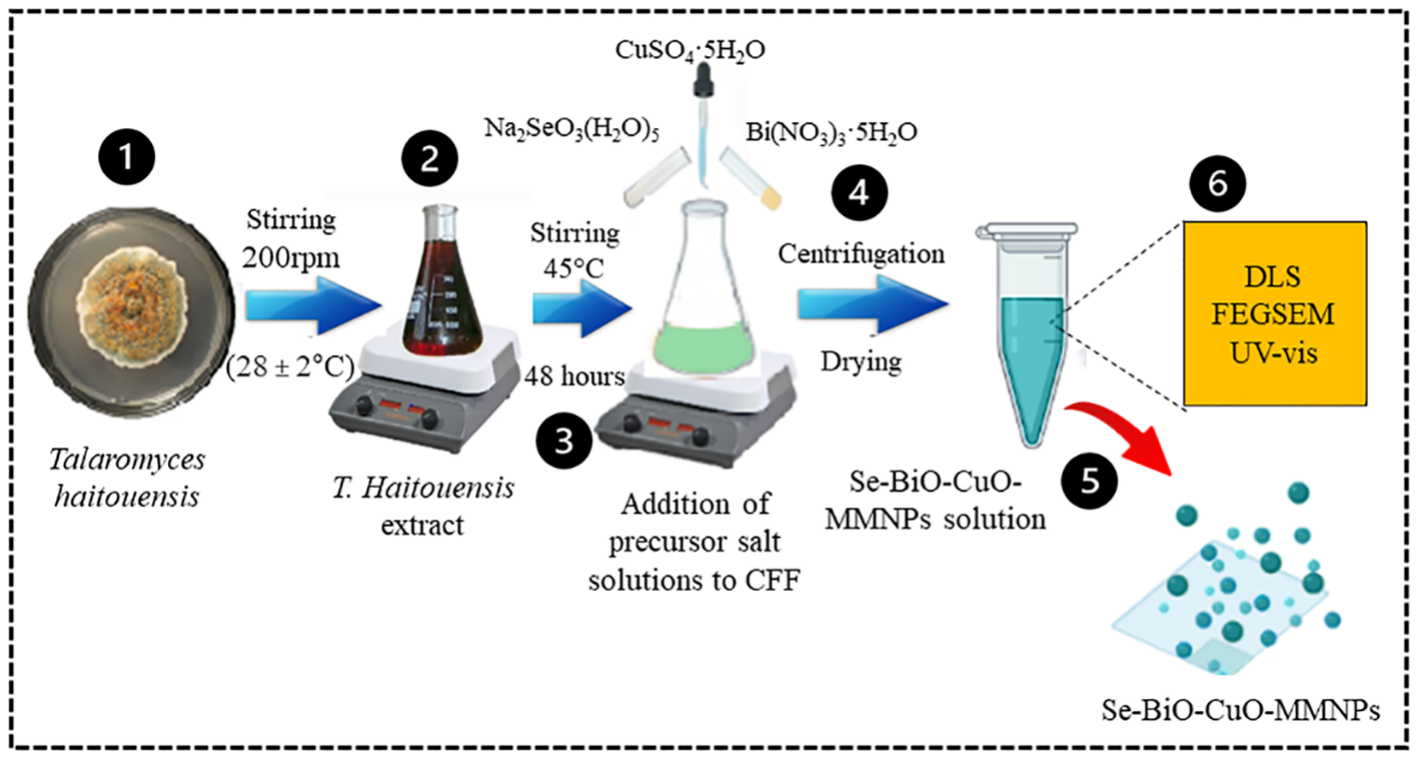
Schematic illustration of *Talaromyces haitouensis* extract-mediated multimetallic nanoparticle synthesis. Isolation and identification of *Talaromyces haitouensis* from rhizospheric soil on SDA. After centrifugation, nanoparticles were allowed to dry at room temperature before being lyophilized and stored in polypropylene tubes. Various spectroscopic techniques were employed to analyze and characterize the synthesized Se-BiO-CuO MMNPs.

Se-BiO-CuO MMNPs were mycosynthesized using *T. haitouensis* cell-free extract as shown in **Scheme 2**. After adding precursor salt solution [CuSO_4_·5H_2_O, Bi(NO_3_)_3_·5H_2_O, and Na_2_SeO_3_(H_2_O)_5_], a dark green color was observed indicating the formation of nanoparticles (Vaseghi et al., 2018).

**Figure 4:**
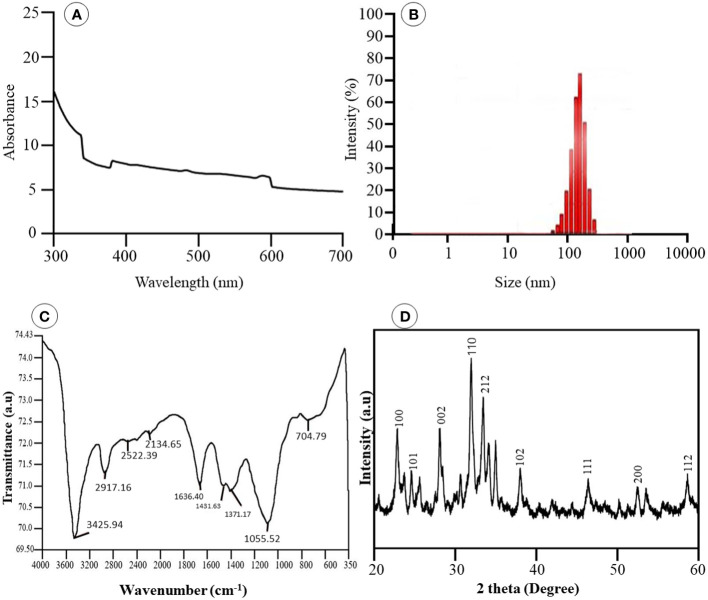
**(A)** UV-vis spectra of Se-BiO-CuO-MMNPs **(B)** Dynamic Light Scattering **(C)** FT-IR analysis of MMNPs **(D)** XRD pattern of Se-BiO-CuO-MMNPs.

UV–visible absorption spectroscopy in the 300- to 700-nm region was used to monitor the formation of Se-BiO-CuO MMNPs. UV–visible spectrum as depicted in **Figure 4A** revealed the presence of the three nanometals in the prescribed range. Predominantly, because of the bands overlapping, the presence of CuONPs, BiONPs, and SeNPs was confirmed by the bands at 570 nm, 376 nm, and 290 nm, respectively (Khodaie and Ghasemi, 2018; Hashem and Salem, 2022; Motakef-Kazemi and Yaqoubi, 2020). The appearance of these peaks, attributed to the surface plasmon resonance band, has been seen for a variety of metal nanoparticles with a wide range of sizes. The particle’s shape, size, and interaction with the medium and the amount of charge transfer between the particle and the medium all play pivotal roles in the formation of surface plasmon resonance. Accordingly, the data obtained prospectively confirm the mycosynthesis of Se-BiO-CuO MMNPs (Muthukrishnan et al., 2015).

**Figure 5:**
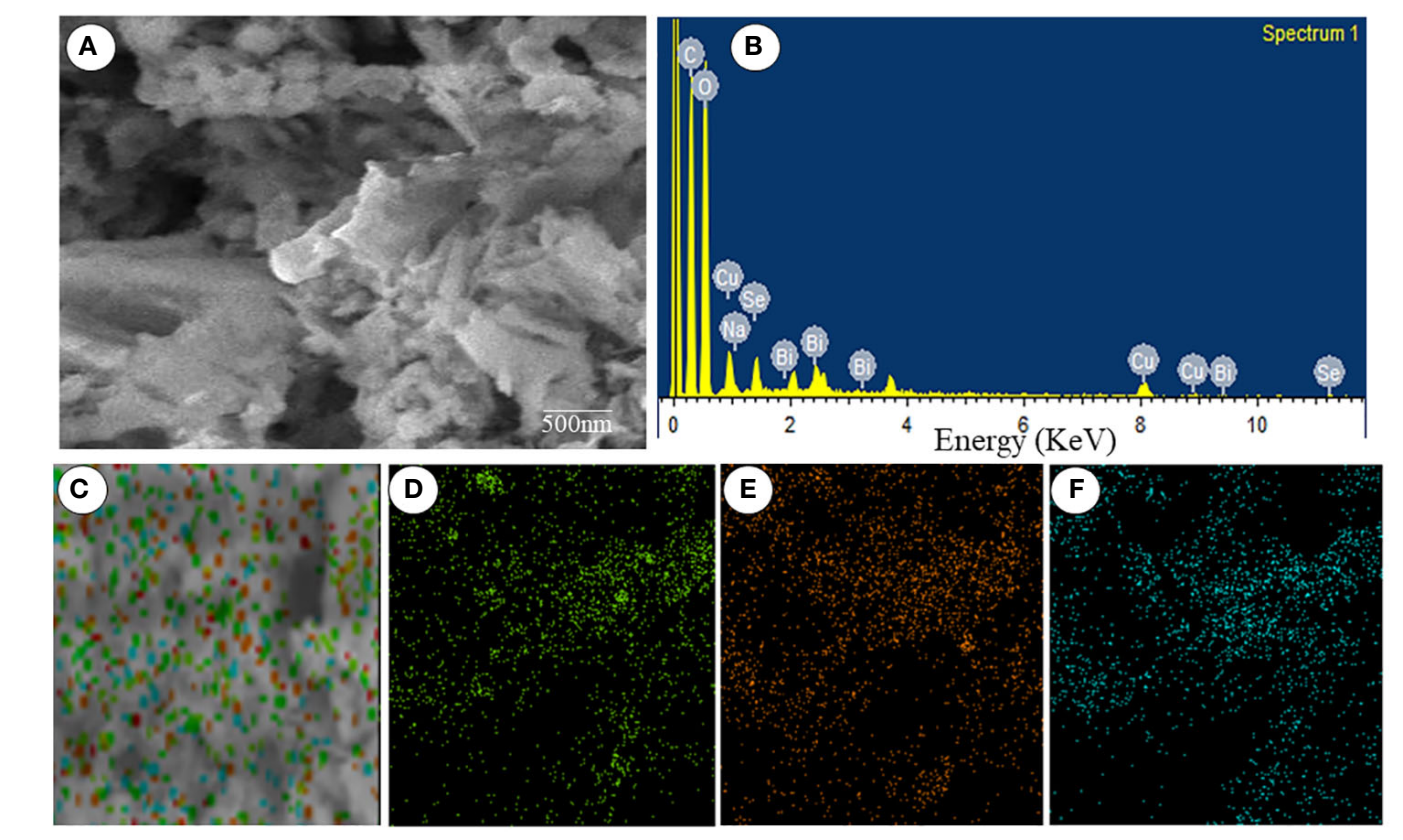
**(A)** Scanning electron microscopy. **(B)** Energy dispersive x-ray spectra. **(C–F)** Elemental mapping spectroscopy images of Se-BiO-CuO multimetallic nanoparticles.

The size distribution of the Se-BiO-CuO MMNPs was determined using the DLS (Microtrac Nanotrac Wave II, PA, USA) technique as shown in **Figure 4B**. The biosynthesized Se-BiO-CuO MMNPs ranged in size from 60 to 500 nm. The *T. haitouensis* extract-mediated Se-BiO-CuO MMNPs had a zeta potential of 13.2 mV, which supports the high repulsion between the particles and consequently boosts the stability of the nanoparticles and prevents agglomeration (Muthuvel et al., 2020). Furthermore, the size of nanoparticles observed by DLS is typically larger than the size obtained by SEM/TEM. The fact is that the DLS measures the hydrodynamic diameter in aqueous solution while others gauge the actual core size of dried nanoparticles (Das et al., 2017). Fourier transform infrared spectroscopy (FT-IR) was used to identify the key functional groups (**Figure 4C**). The O–H stretching of phenolic compounds was attributed to the bond at 3,425.94 cm^−1^, whereas the 2,917.16 cm^−1^ absorption band was attributed to the unsaturated C–H stretching of aldehydes from secondary metabolites. The distinct peak located at 1,636.40 cm^−1^ was unambiguously linked to the N–O asymmetric stretching, indicating the active immersion of nitro compounds respectively. Furthermore, the band remarked at 1,055.52 cm^−1^ could be assigned to the C=C stretching of the broad range alkene group contributing to the synthesis and stability of Se-BiO-CuO MMNPs (Saeed et al., 2019). The x-ray diffraction pattern for CuO, BiO, and Se-NPs was recorded by an x-ray diffractometer in the 2θ range from 20° to 80°. XRD peaks with hkl planes (111), (200), (212), and (102) at 2θ corresponds to CuO-NPs (JCPDS: 78-064 8). The additional peaks of Se-NPS (JCPDS 06-0362) and BiO-NPs (JCPDS card 06-0249) were obtained at the planes (101), (002), (100), (110), and (112), respectively (Saeed et al., 2019; Zhao et al., 2017). The average size of Se-BiO-CuO MMNPs was 66 nm employing Scherer’s equation as shown in **Figure 4D**. The secondary metabolites present in *T. haitouensis* extract are the major contributor to reduced metal ions and stabilized the synthesized Se-BiO-CuO MMNPs (Motakef-Kazemi and Yaqoubi, 2020). SEM coupled with the energy dispersive x-ray (EDX) and mapping were recorded to visualize the texture and diameter of Se-BiO-CuO MMNPs (**Figure 5A**). The amorphous-shaped metallic particles were depicted in the SEM images, which also confirmed that they had aggregated into conventional morphological appearances. The particle also appeared as compact layers with distinct boundaries and edges, which was another important characteristic, and the use of different metals could be the cause of this behavior (Shehabeldine et al., 2023; Motakef-Kazemi and Yaqoubi, 2020; Alagesan and Venugopal, 2019). SEM dimensions revealed that, on average, Se-BiO-CuO MMNPs were approximately 65–80 nm in size.

Mapping microscopy examination revealed the presence of selenium, bismuth, and copper as parts of the Se-BiO-CuO MMNPs, and their complementary distribution was particularly attractive as demonstrated in **Figures 5C–F**. The findings demonstrated a distinct overlap between the selenium, bismuth, and copper in the selected analyzed field, supporting the formation of MMNPs. Similar types of mapping images of Ag–Fe bimetallic nanoparticles have already been reported in the literature (Padilla-Cruz et al., 2021). Moreover, the elemental spectrum of the Se-BiO-CuO MMNP sample was also obtained by additional investigation employing energy-dispersive x-ray spectroscopy (**Figure 5B**), which confirms the synthesis of MMNPs.

### Antibacterial potential of multimetallic nanoparticles

**Figure 6:**
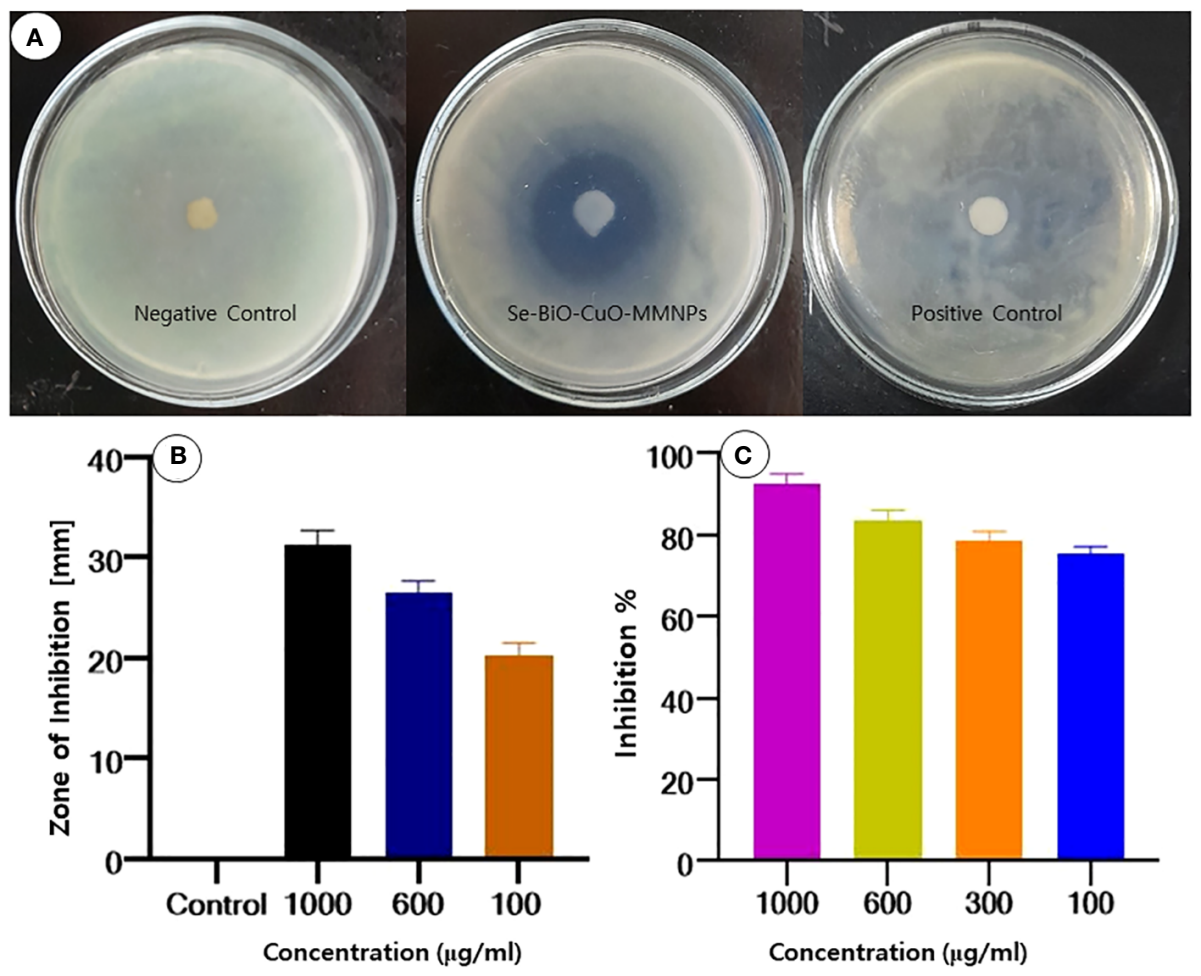
**(A, B)** Zone of inhibition of Se-BiO-CuO MMNPs against ESBL *E coli*. **(C)** MIC growth percentage inhibition of *E coli*.

In an endeavor to evade the effects of antimicrobial drugs, antimicrobial resistance is a natural, intrinsic phenomenon that can also be acquired or transferred. Owing to their natural intrinsic structural or functional characteristics, bacterial species can withstand or resist the effect of antibiotics (Li, 2016). Nanomaterials are of growing interest for researchers to overcome bacterial resistance as they are considered magic bullets (Khan et al., 2016). Because of the synergistic impact produced by different metals, MMNPs can be used as ideal nano antibiotics to combat antimicrobial agents (Basavegowda and Baek, 2021). The disc diffusion approach was used to assess the antibacterial activity of biogenic Se-BiO-CuO MMNPs to acquire some preliminary insight into multidrug-resistant ESBL *E. coli* strain. In this work, it was demonstrated that the synthesized Se-BiO-CuO MMNPs exhibited a stronger inhibitory effect for *E. coli*. The maximum zones of inhibition were 31 ± 0.3 mm at 1,000 μg/mL, whereas the minimum zones of inhibition were 18 ± 0.5 mm at 100 μg/mL as depicted in **Figures 6A, B**. The results indicated that Se-BiO-CuO MMNPs demonstrated dose-dependent biological activities. According to the literature, the higher the concentration of nanoparticles, the higher the inhibitory effect against drug resistance pathogens (Rajivgandhi et al., 2019a). MMNPs were revealed as better antimicrobial agents than monometallic nanoparticles and showed scatter behavior on the surfaces of bacterial cells, causing morphological changes as well as cell respiration and permeability, which led to cell death (Zhao et al., 2020; Al-Haddad et al., 2020; Rajivgandhi et al., 2019c).

The MIC percentage of Se-BiO-CuO MMNPs for the aforementioned microorganism was also established as depicted in **Figure 6C**. Se-BiO-CuO MMNPs MIC ranged from 1,000 to 100 μg/mL. These trends in the MIC data are consistent with findings from earlier research (Wu et al., 2020; Srivastava and Mukhopadhyay, 2015; Das et al., 2020). The growth percentage inhibition graph depicted that the interaction of *E. coli* with the synthesized Se-BiO-CuO MMNPs shows maximum inhibition at 1,000 μg (90%–91% inhibition). The results revealed that concentrations of 600 and 300 μg/mL of Se-BiO-CuO MMNPs had the greatest inhibitory effects, reaching approximately 80%–85%. The MIC for Se-BiO-CuO MMNPs against *E. coli* was determined to be 100 μg/mL, which resulted in a 75% suppression of bacterial growth. The ANOVA-Tukey test revealed a statistically significant difference between different concentrations of the Se-BiO-CuO MMNPs (*p* < 0.05). The graph of *E. coli* growth inhibition percentage after Se-BiO-CuO MMNPs treatment showed a positive association, showing that higher nanoparticle concentrations were correlated with larger bacterial growth inhibition percentages. According to the literature, a reduction in bacterial growth of 0%–20% shows no inhibitory effect, a reduction in the growth of 20%–50% indicates mild inhibition, a reduction in the growth of 50%–70% indicates significant inhibition, and a reduction in growth of more than 70% indicates potent suppression of bacterial growth (Ghorbani et al., 2020). These findings are consistent with the rod-like shape destruction of the *E. coli* strain shown by SEM images. Smaller nanoparticles frequently have a large surface area, which makes it easier for them to contact with the bacterial cell membrane and may eventually alter fundamental processes like penetrability and cell respiration, which can result in cell death (Gold et al., 2018).

### Antibacterial mechanism of action of nanomaterials: unveiling the battle against bacterial infections

**Figure 7:**
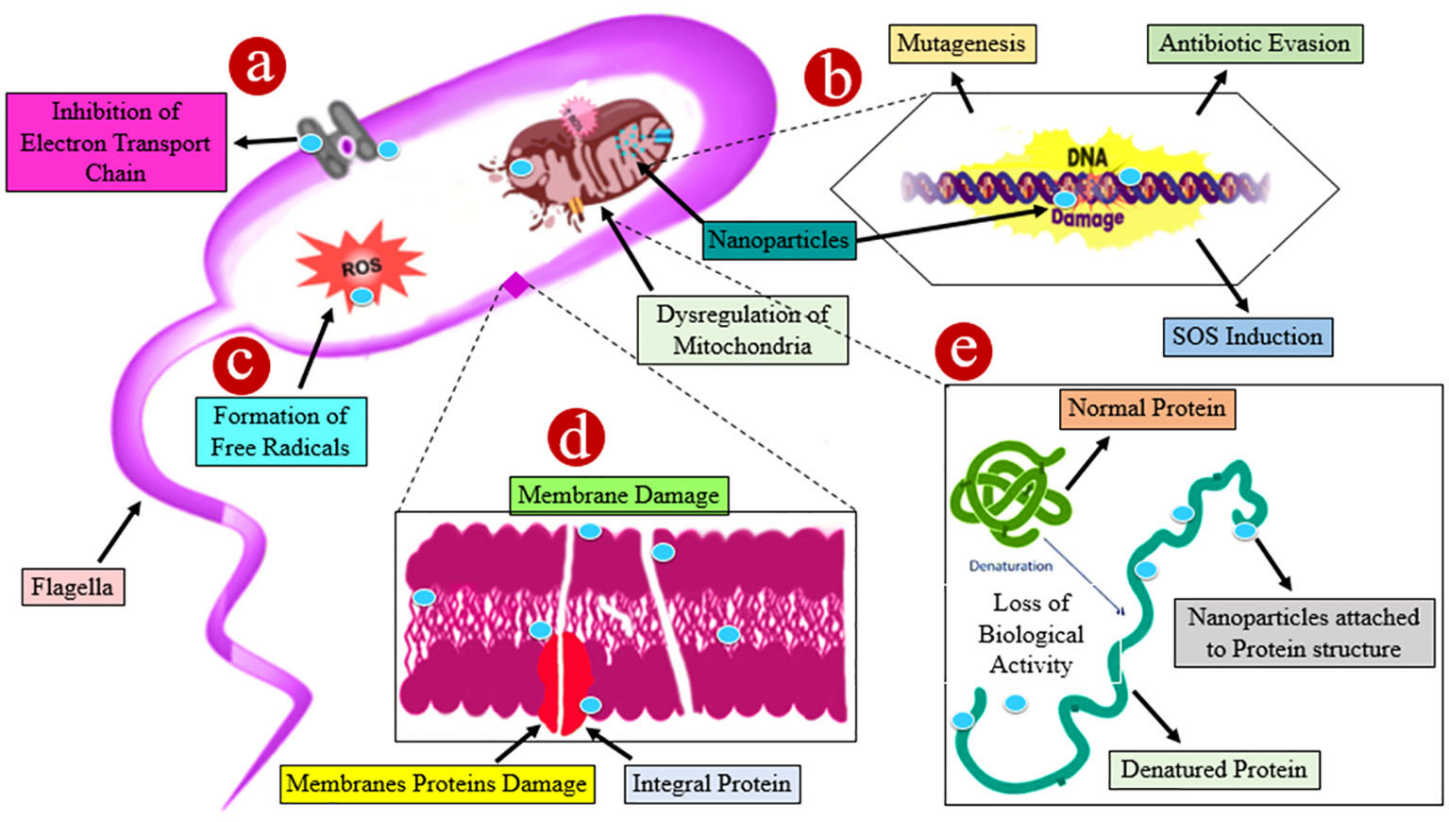
A schematic illustration of numerous probable processes involved in the antibacterial action of different nanoparticles. **(A)** The accumulation of nanoparticles leading to ions damages the cell wall by spawning holes, resulting in the discharge of intracellular constituents and the dwindling of the bacterial cell. **(B)** Nanoparticles have a genotoxic impact, which destroys deoxyribonucleic acid. **(C)** Damage is caused by the interaction of nanoparticles with bacterial membranes owing to the formation of reactive oxygen species (ROS). **(D)** Nanoparticles enhance the transcript levels of stress response genes leading to the development of oxidative stress. **(E)** Protein denaturation occurs as nanoparticles interact with the sulfhydryl group. All of the aforementioned approaches resulted in cell death.

The worldwide quest for new antimicrobial magic bullets is intensifying and is becoming necessary since conventional medications and antibacterial treatments occasionally fail to remove resistant bacteria and biofilms. According to several studies, nanomaterials have shown promising outcomes in the fight against antimicrobial drug resistance (Munir et al., 2020). Nanoparticles exert their antibacterial characteristics through multiple bactericidal mechanisms as depicted in **Figure 7**. Some of these are direct interaction with bacterial cell walls, preventing biofilm emergence and the development of ROS, among others. MMNPs can be extremely effective against biofilm-forming multidrug-resistant bacterial strains because they lack the same mechanisms of action as conventional antibiotics (Basavegowda and Baek, 2021). When compared to small-molecule medications, nanomaterials have advantages in fighting bacteria that are resistant to antibiotics because they can target these biological properties in a variety of ways (Baptista et al., 2018; Hejazy et al., 2018).

### Bacterial cell membrane disruption by Se-BiO-CuO MMNPs

**Figure 8:**
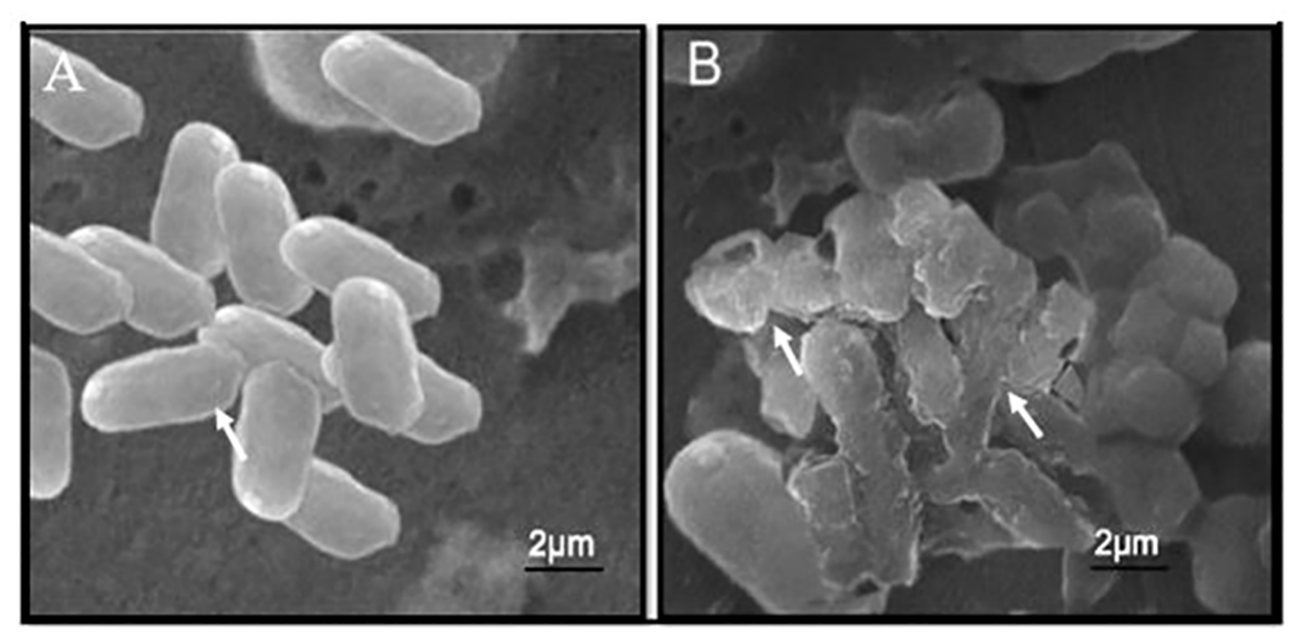
SEM micrographic images of untreated *E. coli* cells **(A)** and *E. coli* cells with Se-BiO-CuO multimetallic nanoparticles after 24 h of incubation **(B)**.

The SEM images revealed that the bacterial cells showed significant morphological alteration, upon exposure to synthesized Se-BiO-CuO MMNPs. Bacteria adjust their cell morphologies to enhance their resistance to distinct antimicrobial agents that target different biological components. As demonstrated in **Figure 8A**, untreated *E. coli* cells had a normal, rod-shaped appearance; their cytoplasm was homogeneous and their morphology, membrane, and wall structures showed integrity. However, damage findings were quite high in cells treated with nanocomposite; cell deaths and shape irregularity are visible whereas some shrunken cells can be seen in **Figure 8B**. Muthuchamy et al. also reported the effects of nanocomposites on the Gram-negative bacterial cell wall (Muthuchamy et al., 2020). The precise antibacterial mechanism of nanoparticles has not been fully elucidated and still requires further investigation. It is speculated that these nanoparticles adhere to the cell membrane, leading to the disruption of the bacterial cell membrane, ROS formation, and inhibition of enzyme synthesis, which, in turn, affects genetic replication (Rajivgandhi et al., 2020).

Moreover, it is also reported that metal ions produced by different nanoparticles that interacted with the negatively charged cell wall entered and interrupted its function and ultimately caused protein denaturation, fragmentation, and deformation of bacterial cells, which ultimately leads to cell death (Asghar and Asghar, 2020; Sanpui et al., 2008). There is less chance of antimicrobial resistance because of the synergistic effects of the combination of two or more metals and multiple target sites. As an alternate approach to combating pathogenic diseases that are resistant to antibiotics, synergistic interactions or combinatorial therapy have drawn more attention (Lee et al., 2019).

### Evaluation of kinetic action

**Figure 9:**
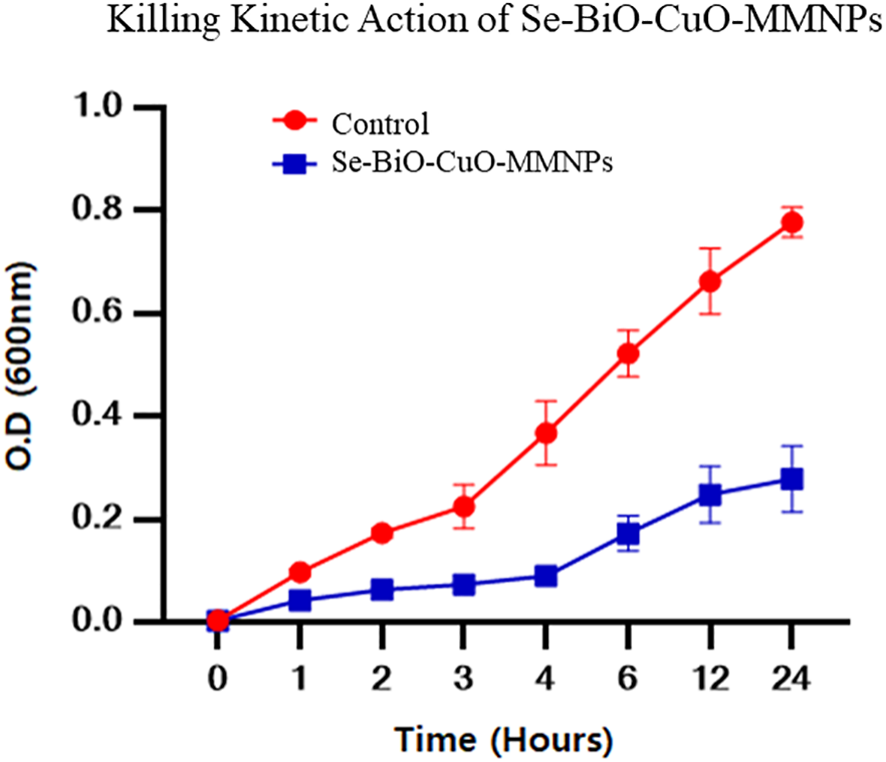
Time-dependent killing kinetic assay of Se-BiO-CuO MMNPs against multidrug-resistant ESBL *E. coli* pathogen. Corresponding MIC was utilized to observe the time effectiveness.

The time-killing test was piloted using the previously reported protocol to better understand the kinetics action of nanoparticles against bacteria (Maruthai et al., 2017). It was reported that the bacteriostatic action of nanoparticles against bacterial cells was directly proportional to the concentration of nanoparticles used (Abishad et al., 2022). Furthermore, it was demonstrated from the results that after 4 h of incubation, Se-BiO-CuO MMNPs at their MIC effectively halted ESBL *E. coli* bacterial growth as depicted in **Figure 9**. Gram-negative bacteria were devastated more quickly because of weaker cell wall composition as compared to Gram-positive bacteria even at low concentrations of antibacterial compounds. This could be attributed to the increased surface area and higher positive surface density, enabling enhanced interaction with negatively charged bacterial cell membranes. This, in turn, leads to increased cell permeability and the penetration of nanoparticles into bacterial cells, ultimately resulting in bacterial cell death (Shehabeldine et al., 2023). The experiment was performed in triplicate.

### Swimming motility assay

**Figure 10:**
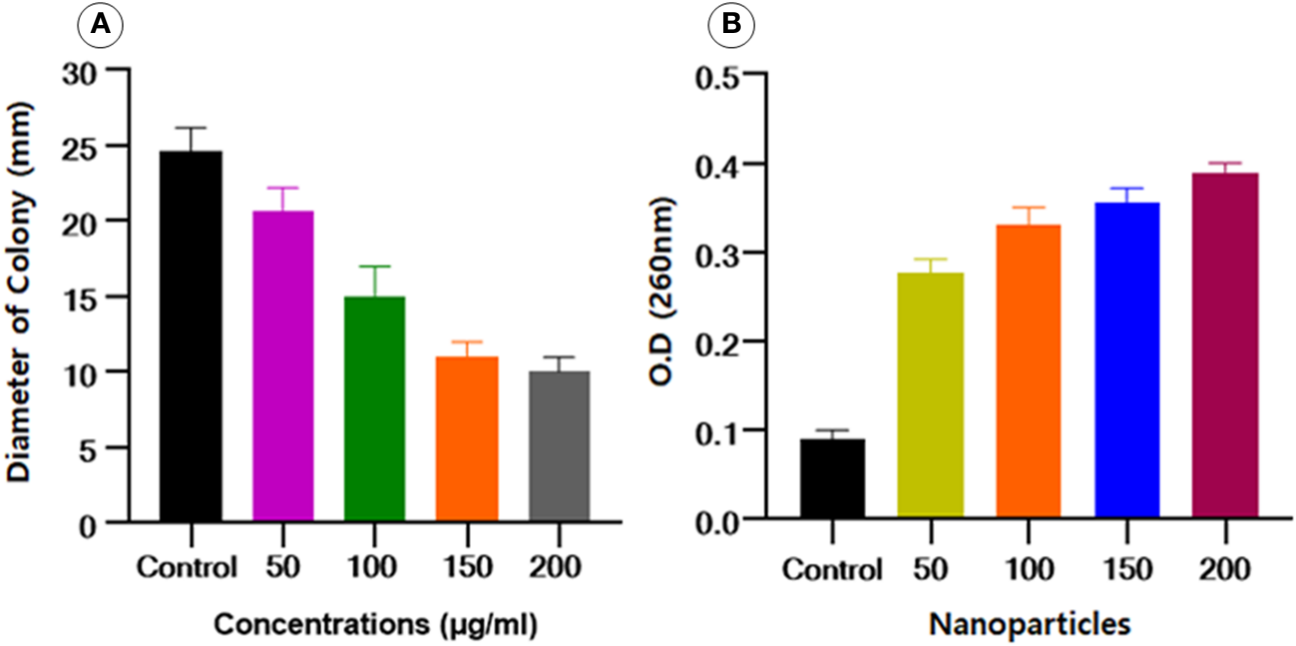
**(A)** Effect of Se-BiO-CuO MMNPs on the swimming motility of *E. coli.* **(B)** *E. coli* bacterial cells’ cytoplasmic efflux release upon exposure to various Se-BiO-CuO MMNPs concentrations (μg/mL) and control (*p* ≤ 0.05).

According to the already reported protocol (Saeki et al., 2021), the impact of Se-BiO-CuO MMNPs at concentration levels of 50 to 200 μg/mL on the diameter-based swimming motility of ESBL *E. coli* was examined. The results of this study indicated that the Se-BiO-CuO MMNPs had a potent inhibitory effect on ESBL *E. coli* bacterial swimming as illustrated in **Figure 10A**. The lowering of bacterial swimming ability is correlated with the kind and concentration of nanoparticles in addition to the antibacterial effect. Therefore, we conjectured that by preventing bacterial swimming movement, nanoparticles will more effectively combat bacterial pathogenicity. Our results are related to Cheng et al. (2020), who found that silver nanoparticles can reduce the swimming motility of *R. solanacearum* strain YY06.

### Cytoplasmic efflux analysis

Intracellular component leakage was liberated from the cell when the cell membranes burst, which could be seen by using a spectrophotometer at an optical density of 260 nm. Se-BiO-CuO MMNPs at various doses significantly altered the cytoplasmic efflux release of the ESBL *E. coli* strain as shown in **Figure 10B**. The cell membrane was significantly destroyed as seen by the dramatic increase in OD values over time as compared to controls. After 10 h of incubation for all concentrations used, the absorbance increased in a dose-dependent way and reached its maximum, indicating that the cell membrane was utterly damaged. Our findings are similar to the already reported study (Chen et al., 2013).

### Biofilm inhibition assay

**Figure 11:**
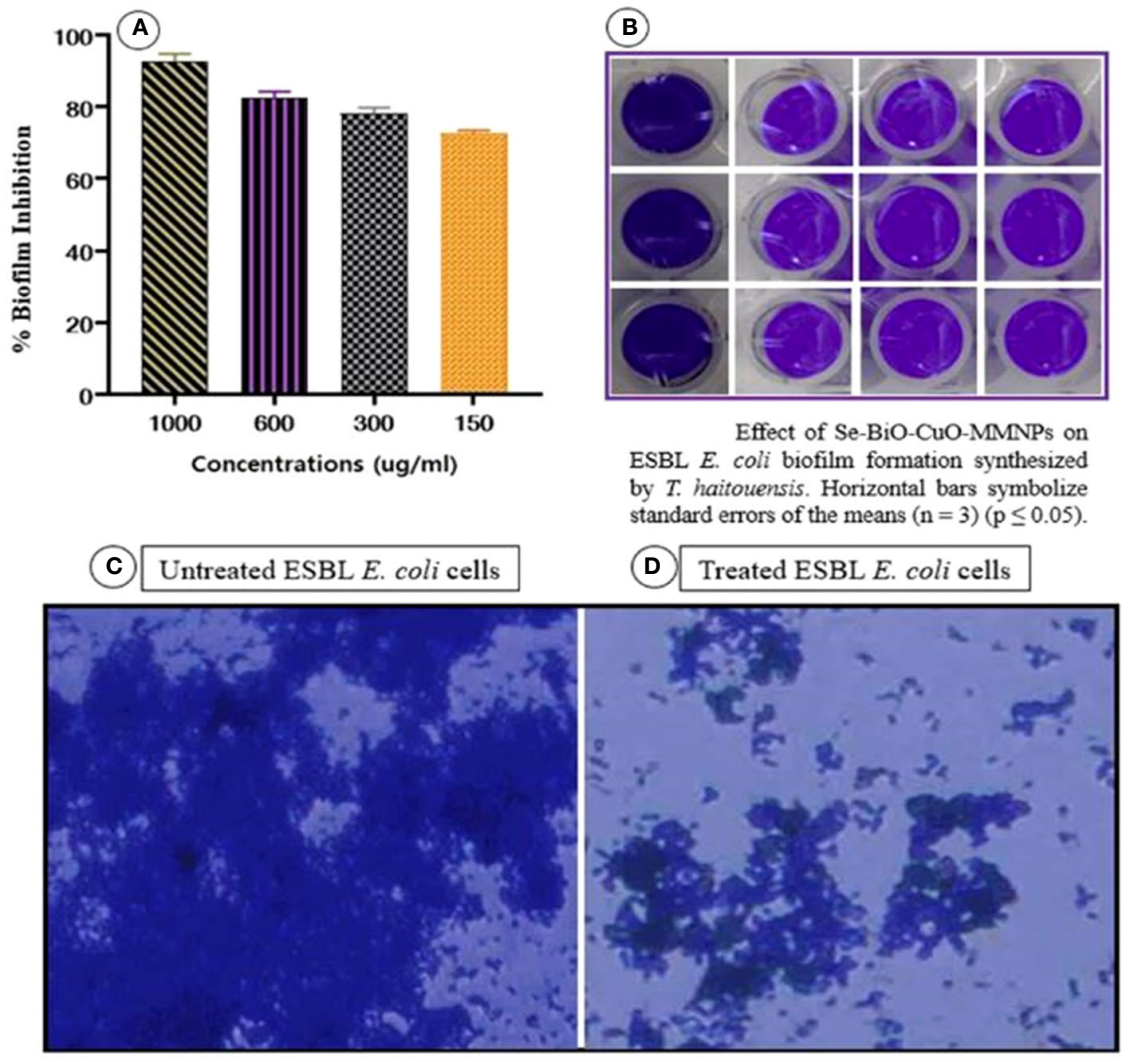
**(A, B)** Biofilm inhibition percentage of Se-BiO-CuO MMNPs against *E. coli* biofilm-forming bacterial strain and **(C, D)** anti-biofilm images of untreated and treated *E. coli* cells (*p* < 0.05).

The biofilm assay was performed to assess the impact of Se-BiO-CuO MMNPs synthesized from *T. haitouensis* extract on the formation of *E. coli* biofilm. The ability of the Se-BiO-CuO MMNPs to suppress biofilm development was confirmed with a crystal violet staining and absorbance was measured at 595 nm using a microplate reader (Model FL ×800; Biotek, VT, USA). The percentage of biofilm inhibition at various Se-BiO-CuO MMNP concentrations (1,000, 600, 300, and 150 μg/mL) is shown in **Figures 11A, B**. The results obtained in the microtiter plates show that the highest inhibition percentage was 89.4% with 1,000 μg/mL Se-BiO-CuO MMNPs, followed by 600 μg/mL Se-BiO-CuO MMNPs (84.2%). A concentration of 300 μg/mL resulted in an inhibition percentage of 80.5%. At the MIC value of 150 μg/mL, Se-BiO-CuO MMNPs inhibited biofilm development by 74.8%. In this study, Se-BiO-CuO MMNPs exhibited significant anti-biofilm efficacy against *E. coli* biofilm.

The change in the treated and untreated ESBL *E. coli* biofilm architecture was depicted using cell imager (Evos^®^R FL Cell Imaging System; Thermo Fisher Scientific, MA, USA) as shown in **Figures 11C, D**. The micrographs clearly showed that Se-BiO-CuO MMNPs affected the density of the biofilm, which is consistent with previous research (Qais et al., 2021). Our findings support those of Vinotha et al., who reported that ZnO nanoparticles can reduce the thickness of *E. faecalis*, *S. aureus*, *P. aeruginosa*, and *P. vulgaris* colonies (Vinotha et al., 2020; Siddhardha et al., 2020).

### Biocompatibility analysis

**Figure 12:**
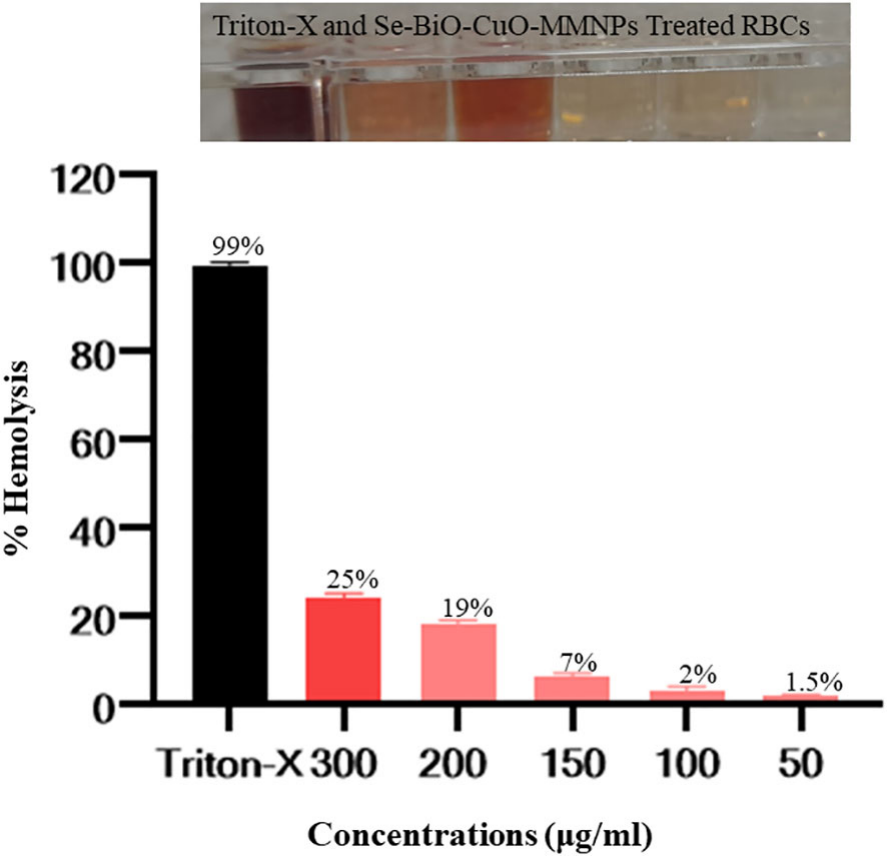
Assaying the biocompatibility of Se-BiO-CuO MMNPs. The *y*-axis displays the hemolysis percentage, and the *x*-axis shows the concentration. In contrast to the Triton X-100 (as a positive control), which displayed complete hemolysis, Se-BiO-CuO MMNPs depicted concentration-dependent hemolytic activity.

In recent years, with the advancement of nanoparticle-based diagnosis, the issue of potential toxicity has gained prominence. It is imperative to examine the toxicity of nanomaterials on blood, most particularly RBCs. Nanoparticles are known to induce membrane destruction and cell demise when exposed to erythrocytes. Se-BiO-CuO MMNPs showed concentration-dependent hemolytic activity. Hemolysis was observed to be minimal at the lowest concentration of 50 μg/mL with a recorded percentage of 1.5%. In contrast, the highest concentration of 300 μg/mL resulted in a higher percentage of hemolysis, reaching 25%. The hemolysis assay verdicts indicate that the green synthesized Se-BiO-CuO MMNPs at a concentration of 100 µg/mL displayed hemolysis as shown in **Figure 12**, which is within the biocompatible range (Li et al., 2012). The results of the hemolysis study showed promising blood biocompatibility and, thus, might be considered for *in vivo* biomedical applications.

## Conclusion

In this study, *T. haitouensis* extract was used for the synthesis of Se-BiO-CuO MMNPs. To the best of our knowledge, we reported for the first time *T. haitouensis-*mediated synthesis of Se-BiO-CuO MMNPs. Physiochemical characterizations confirmed the amorphous-shaped Se-BiO-CuO MMNPs with an average size of approximately 66–80 nm. *E. coli* is the dominant and leading cause of bacteremia. The double-disc synergistic test confirms the production of ESBL-positive *E. coli*. The synthesized Se-BiO-CuO MMNPs exhibit significant antibacterial activity with an MIC of 100 μg/mL against the ESBL *E. coli* strain. Moreover, Se-BiO-CuO MMNPs displayed strong antibiofilm action. SEM images also highlighted the bacterial structure disruption. Furthermore, it was demonstrated from the results that after 4 h of incubation, Se-BiO-CuO MMNPs effectively ceased ESBL *E. coli* bacterial growth. The cell membrane disruption and intracellular component leakage were also observed by cytoplasmic efflux assay. The results of the hemolysis study showed that Se-BiO-CuO MMNPs showed hemolysis (2%) within biocompatible range. These results imply that green synthesized MMNPs act as promising therapeutic agents to combat bacterial infections including those caused by the ESBL *E. coli* strain. It was also observed that MMNPs showed promising results in mitigating bacterial infections. Consequently, additional research endeavors are needed to validate the antibacterial effects of Se-BiO-CuO MMNPs against other ESBL-positive pathogens for *in vivo* research.

## Data availability statement

The original contributions presented in the study are included in the article/**Supplementary Material**, further inquiries can be directed to the corresponding author/s.

## Author contributions

BU: Conceptualization, Methodology, Supervision, Writing – original draft. RR: Formal analysis, Writing – original draft. AR: Funding acquisition, Investigation, Writing – review & editing. RB: Resources, Validation, Writing – review & editing. NA: Resources, Validation, Writing – original draft.
