# Supplementary figures and images for: Fungus-mediated synthesis of Se-BiO-CuO multimetallic nanoparticles as a potential alternative antimicrobial against ESBL-producing Escherichia coli of veterinary origin
Source: Front Cell Infect Microbiol. 2024 Mar 22;14:1301351. doi: 10.3389/fcimb.2024.1301351 (PMC11037251; doi:10.3389/fcimb.2024.1301351)

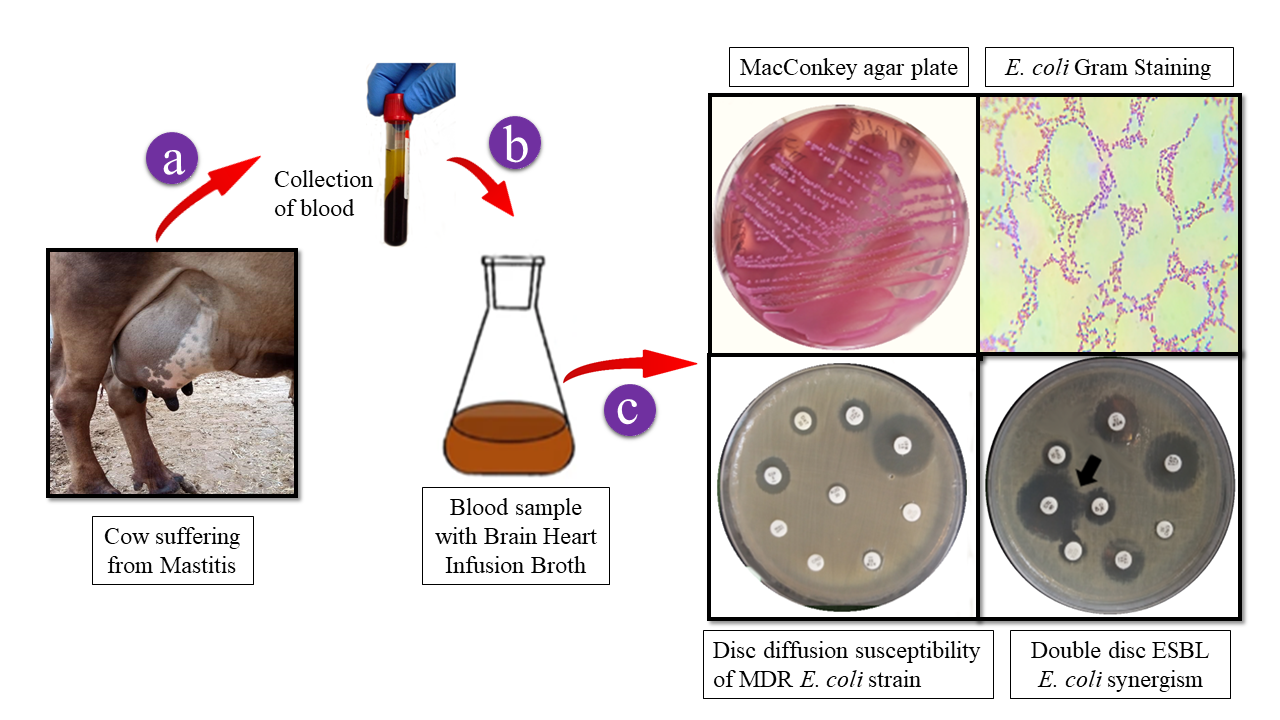

Supplement: Supplementary file 1 [file Image_1.tif]

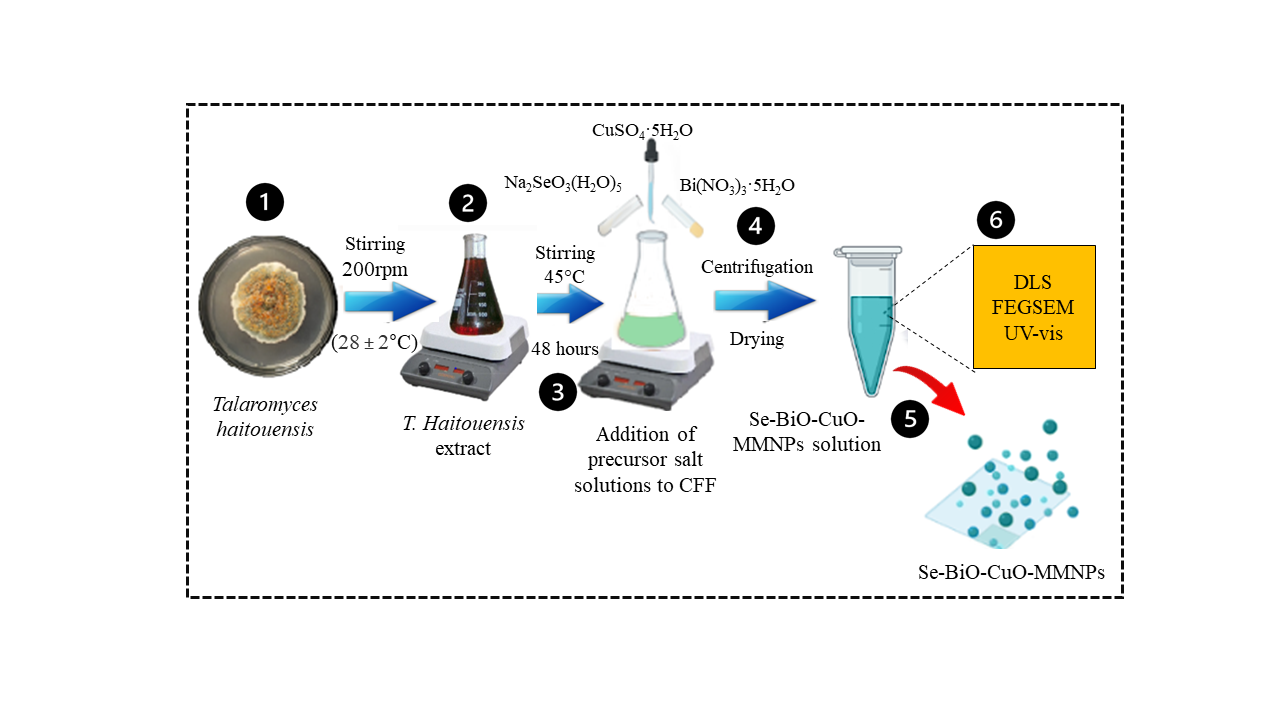

Supplement: Supplementary file 2 [file Image_2.tif]
